# Identification of movement synchrony: Validation of windowed cross-lagged correlation and -regression with peak-picking algorithm

**DOI:** 10.1371/journal.pone.0211494

**Published:** 2019-02-11

**Authors:** Désirée Schoenherr, Jane Paulick, Bernhard M. Strauss, Anne-Katharina Deisenhofer, Brian Schwartz, Julian A. Rubel, Wolfgang Lutz, Ulrich Stangier, Uwe Altmann

**Affiliations:** 1 Jena University Hospital, Institute of Psychosocial Medicine and Psychotherapy, Jena, Germany; 2 Trier University, Department of Clinical Psychology and Psychotherapy, Trier, Germany; 3 Goethe University Frankfurt/Main, Department of Clinical Psychology and Psychotherapy, Frankfurt/Main, Germany; The Hong Kong Polytechnic University, HONG KONG

## Abstract

In psychotherapy, movement synchrony seems to be associated with higher patient satisfaction and treatment outcome. However, it remains unclear whether movement synchrony rated by humans and movement synchrony identified by automated methods reflect the same construct. To address this issue, video sequences showing movement synchrony of patients and therapists (N = 10) or not (N = 10), were analyzed using motion energy analysis. Three different synchrony conditions with varying levels of complexity (naturally embedded, naturally isolated, and artificial) were generated for time series analysis with windowed cross-lagged correlation/ -regression (WCLC, WCLR). The concordance of ratings (human rating vs. automatic assessment) was computed for 600 different parameter configurations of the WCLC/WCLR to identify the parameter settings that measure movement synchrony best. A parameter configuration was rated as having a good identification rate if it yields high concordance with human-rated intervals (Cohen’s kappa) and a low amount of over-identified data points. Results indicate that 76 configurations had a good identification rate (IR) in the least complex condition (artificial). Two had an acceptable IR with regard to the naturally isolated condition. Concordance was low with regard to the most complex (naturally embedded) condition. A valid identification of movement synchrony strongly depends on parameter configuration and goes beyond the identification of synchrony by human raters. Differences between human-rated synchrony and nonverbal synchrony measured by algorithms are discussed.

## Introduction

The term *nonverbal behavior* is used to describe various behaviors such as gaze, gestures, facial expressions, body postures and movements [1]. The investigation of *nonverbal synchrony* within a dyad has gained increased consideration within areas of research such as physician-patient interactions [2], psychotherapist-patient interactions [3–5], mother-child communication [6–8], human-machine interactions [9], interactions within friendships [10, 11] and courtship behavior [12]. Nonverbal synchrony refers to nonverbal behaviors of interacting individuals that are connected to each other on a temporal level [11, 13]. In contrast to posture mirroring as showing in the same static posture, movement synchrony as a specific type of nonverbal synchrony, refers to the dynamic aspect, which is the temporal connection between motions of the interacting persons, independent of a particular body part or direction [14]. This nonverbal synchrony can be observed in short sequences that we call synchronization intervals [10]. In psychotherapy context, movement synchrony would be observed, for example, if the therapist is nodding and the patient is changing his/her body position with a short time delay or when both interaction partners are nodding simultaneously.

In comparison to human ratings, methods that automatically record nonverbal behavior and determine nonverbal synchrony are economic, less time-consuming and require fewer resources. A current method that automatically generates time series that represent movements of individual persons in a video is motion energy analysis (MEA) [5, 6, 11]. If multiple persons have been recorded, time series can be computed for each person within a video by determining a region of interest (ROI). After computation, motion energy time series can be used to identify movement synchrony. For this purpose, typical automated methods such as cross-recurrence quantification analyses, spectral analyses, or correlative and regressive time series analyses methods are applied (TSAM) [15].

**Fig 1:**
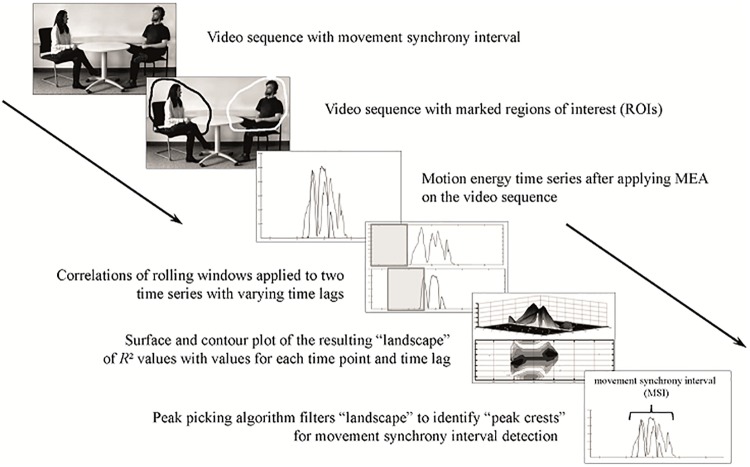
Computational steps from video sequence to identification of a movement synchrony interval.

In the social and behavioral sciences, the use of correlative and regressive TSAM predominantly prevails. Two prominent examples of correlative and regressive TSAM are windowed cross-lagged correlation (WCLC) [16] and windowed cross-lagged regression (WCLR) [10, 11]. The application of TSAM on movement time series is based on a movement synchrony definition of interacting partners’ synchronous or time-lagged synchronous movements. The underlying methodical principle can be described as follows: The correlation (or regression) between the first segment of the time series of person A and the first segment of the time series of person B is calculated. Next, this local association is tested for significance (e.g., *R*^2^-difference-test). Afterwards, the segment of the time series of person B is shifted and procedure is repeated until the entire time series and all reasonable time lags between A and B have been tested. The result of such windowed cross-lagged correlation (WCLC) or windowed cross-lagged regression (WCLR) is a “landscape” (*R*^2^-matrix), which shows at which time point the behavior of A is significantly associated with the simultaneous or time-lagged behavior of B. Subsequently, different algorithms can be used to analyze this matrix and obtain a global synchrony score. For example, Altmann [10], Altmann [11] used a modification of Boker et al.’s (2002) peak-picking algorithm to determine synchronization intervals. These intervals were characterized by the time lag and a specific start and end point. Different indices may be calculated to obtain a global score, for example, the averaged strength of the association (averaged over time and different time lags) as used by Ramseyer and Tschacher [5]. We abbreviate this method and the corresponding index with WCLC_S_ (subscript S stands for strength). Another possibility is the WCLC_F_ (subscript F stands for frequency) or the WCLR, where the frequency of synchrony is calculated as the ratio between synchronized time and total time (for more details, see Schoenherr, Paulick [17]). Fig 1 illustrated the computational steps for the movement synchrony identification.

Previous studies have shown that, based on identical time series, different correlative and regressive TSAM lead to different synchrony indices [11, 17]. Additionally, calculating synchrony for different baseline-surrogate datasets with varying parameter settings (e.g., window size = bandwidth) lead to different results [18]. This indicates that the computed value of synchrony strongly depends on the algorithm *and* parameter settings applied. However, to date, there is no consensus on which parameter settings lead to optimal results. Table 1 displays examples of the use of WCLC, WCLR and related procedures as well as the selected parameter settings.

**Table 1:** Overview of applied parameter setting.

	Smoothing procedure	Transformation	Method	Bandwidth	Step	Measures per second
Altmann [10], Altmann [11]	Smoothing splines (λ = .9995)	Anscombe transformed	WCLR, WCLC_F_	100 frames (ca. 4 sec)	2 frames (0.08 sec)	24
Ashenfelter, Boker [19]	-	-	WCLC	160 frames (2 sec)	10 frames (0.125 sec)	80
Bilakhia, Petridis [20]	-	-	WCLC	-		58
Boker, Rotondo [16]	-	-	WCLC	320 frames (4 sec)	8 frames (0.1 sec)	80
Boker and Rotondo [21]	-	-	WCLC	160 frames (2 sec)		80
Bozkurt, Yemez [22]	-	-	WCLC adapted (CCA)	180 frames (6 sec)		30
Campbell [23]	Low pass filtered		WCC	1 frame		-
Delaherche and Chetouani [15]	-	Ramseyer’s normalization procedure	WCLC	1 sec		-
Paulick, Deisenhofer [3]	Moving average 10	z-transformed	WCLC_S_	60 frames (1 min)	60 frames (1 min)	10
Messinger, Mahoor [7]	-	z-transformed	WCLC	3 sec	-	Ca. 30
Michelet, Karp [24]	-	-	WCC	75 frames	-	25
Nagaoka and Komori [25]	-	Box-Cox transformed	WCC	18000 frames (10 min)	150 frames (5 sec)	30
Paxton and Dale [26]	Butterworth low-pass filter		CLC			8
Ramseyer and Tschacher [5], Ramseyer and Tschacher [27], Ramseyer and Tschacher [28]	Moving average 10	z-transformed	WCLC_S_	600 frames (1 min)	1 frame (0.1 sec)	10
Sun, Nijholt [29]	-	-	WCC	Between 20–280 frames	-	-
Tschacher, Rees [30]	Moving average 10	z-transformed	WCLC_S_	300 frames (30 sec)	0.1 sec	10
Tronick, Als [31]	-	-	WCC	10 frames (10 sec)	-	1
Yang, Wang [32]	-	-	WCLC	Between 64–256 frames (0.64–2.56 sec) Best: 128 frames (1.28 sec)	32 frames (0.32 sec)	100
Watanabe [6]	-	-	WCLC	480 frames (8 sec)	-	60

Note. Subscript letters indicate the type of global output parameter (for details see [17]). WCLC: windowed cross-lagged correlation, WCC: windowed cross-correlation, CLC: cross-lagged correlation, WCLR: windowed cross-lagged regression, CCA: canonical correlation analysis, λ refers to the roughness penalty parameter, ‘-‘ indicates that information was not reported.

### Smoothing procedure

**Fig 2:**
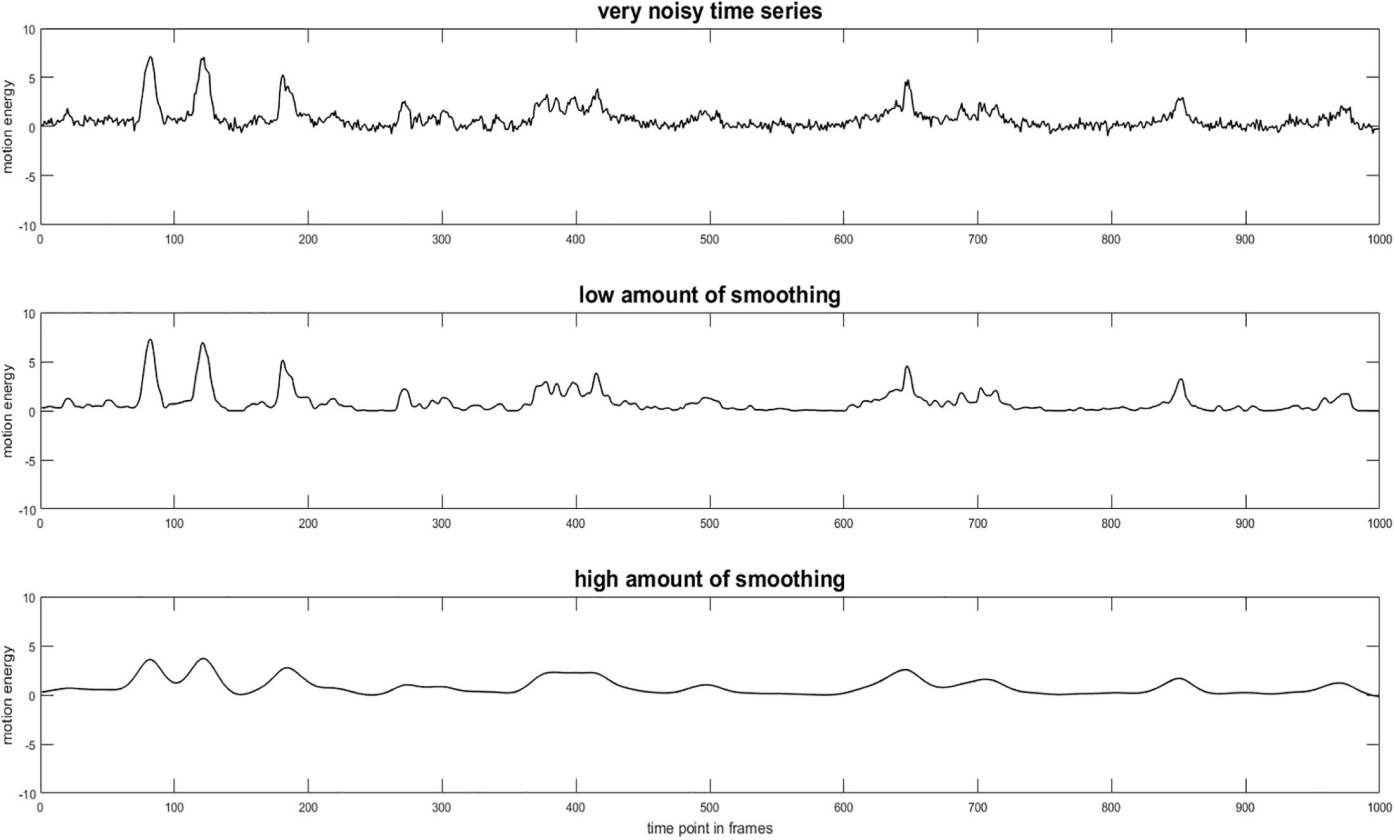
Different amounts of smoothing applied to motion energy time series (METS).

In order to eliminate video noise, many authors recommend smoothing the time series before applying TSAM. Grammer, Honda [33], Paulick, Deisenhofer [3] and Ramseyer and Tschacher [5], Ramseyer and Tschacher [27] used a moving average method. Nagaoka and Komori [25] utilized wavelets. Altmann [10], Altmann [11] recommended smoothing splines and Paxton and Dale [26] used a Butterworth low-pass filter. Apart from the different methods, the critical issue is the amount of smoothing applied. To date, however, it remains unclear which amount of smoothing leads to improved synchrony detection. Fig 2 illustrates the impact of smoothing on the shape of the time series.

### Transformation

**Fig 3:**
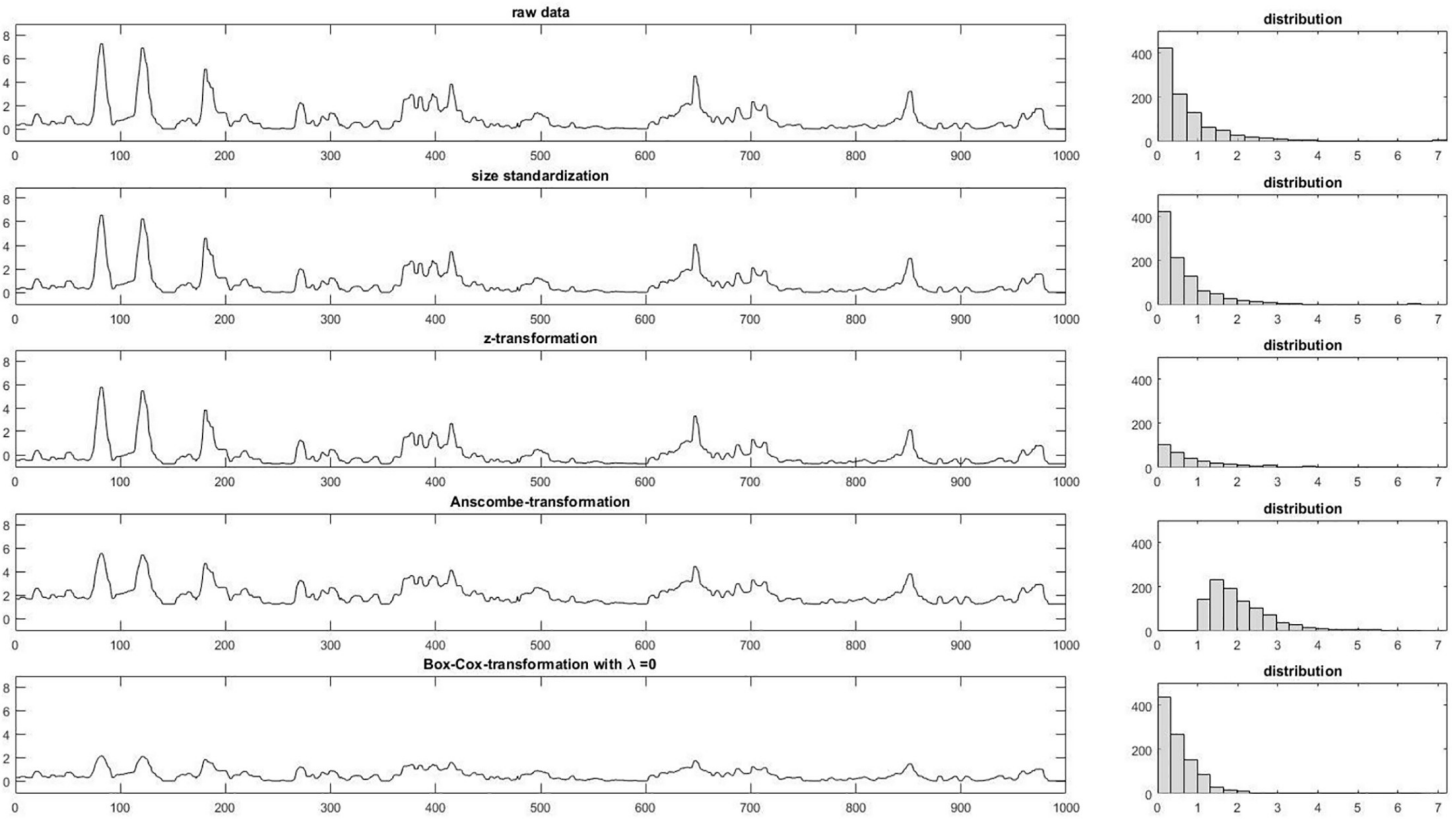
Different transformations and their impact on the distribution of motion energy values assessed with MEA.

Motion energy time series are non-stationary [11]. This means that the variance and the expectation value of the time series are not stable over time. A linear trend or cyclic behavior in both time series under consideration could lead to spurious correlations, which may cause an over-identification of synchronization intervals [10]. Authors have attempted to control non-stationarity by transforming the distribution of values into an approximately normal distribution [11]. By means of an Anscombe transformation [34] or Box-Cox transformation [35], a normal distribution can be approximated. Moreover, these transformations have a variance-stabilizing effect. Nagaoka and Komori [25] used a Box-Cox transformation with the parameter λ = 0, which corresponds to a logarithmic transformation, whereas Altmann [11] recommends the Anscombe transformation. Furthermore, z-standardization is used to standardize the value range of time series from different persons [5, 27]. However, this transformation does not lead to any change of the distribution or variance. Another option to standardize the value range is to transform the motion energy time series (METS) with respect to the ROI size (size standardization—for details see Methods). As a result, values of this linear transformation vary from zero (no motion) to 1 (entire ROI is active). Fig 3 displays the different transformations and their impact on the distribution of values.

### Method

Two methods that show high convergent validity and a local identification of movement synchronization intervals (MSI) are the WCLR and WCLC_F_ from Altmann [10], Altmann [11], [17]. The algorithms are publicly available at GitHub: https://github.com/10101-00001/sync_ident. The output of both methods is a table showing every MSI of the interaction under investigation. This enables us to compare these MSI with intervals rated by human raters. The WCLR was developed for cyclic time series to avoid spurious cross-correlations due to auto-correlation. We used these two methods because they identify synchronization intervals. These intervals may be used to investigate more local events that emerge on a short-time level as alliance ruptures [36] or good moments within therapy. Furthermore, these measures had the highest correlation with an external criteria in the validation study of Schoenherr, Paulick [17].

### Bandwidth

An important parameter in the application of windowed correlative and regressive methods is bandwidth. Bandwidth specifies the length of the examined segment. The choice of bandwidth depends on content and methodological considerations [16]. If the bandwidth is set too long, the detected synchrony may be biased and the true dynamics of the interaction may not be optimally mapped. In contrast, with small bandwidths, it is also possible that synchronization intervals remain undetected. Additionally, if the selected segment is too small, the correlation or regression cannot be reliably determined due to the low number of measured values. Schönbrodt and Perugini [37] recommend a sample size of 250 values for stable parameter estimation with respect to typical correlation analyses. This number, however, is dependent on the true correlation. For high correlations (*r* = .7), the authors found a sample size of 65 to be sufficient. An optimal trade-off for high reliability and high sensitivity has not yet been determined [16]. The choice of the bandwidth is also dependent on the number of values recorded per second. Currently, every researcher chooses the bandwidth with respect to the phenomena under investigation. If one wants to assess global synchrony that is established over period, higher bandwidths (30 seconds and higher) are used [5]; if the phenomena under investigation are local synchrony intervals, smaller bandwidths (2.5 seconds: Altmann [10]) are applied.

With respect to the problem of non-stationarity, Boker, Rotondo [16] proposed that the assumption of stationarity can be made locally within segments. Therefore, using windows may also be beneficial for statistical reasons.

### Spurious correlations

Various approaches can be used to control for coincidentally identified synchronization intervals. Ramseyer and Tschacher [38] built on the concept of pseudosynchrony by Bernieri, Reznick [39]. They created an artificial database of interactions by means of random permutations of the segments of the real time series. In doing so, they created an empirical distribution to determine a cut-off value to discriminate between pseudosynchrony and genuine synchrony. Louwerse, Dale [40] also built surrogate pairs to compare genuine synchrony with pseudosynchrony by using random permutations of data points. Recently, Moulder, Boker [18] compared different methods of surrogate dataset generation (data shuffling, segment shuffling, data sliding, participant shuffling) showing that not every baseline is equally conservative. Additionally, there seems to be an interaction with parameters chosen for applying the WCLC. In contrast to baselines which are based on surrogate datasets, Gottman and Ringland [41] and Altmann [10], Altmann [11] propose a parametrical approach to test genuine synchrony against randomly occurring synchrony, whereby the distribution of the proof statistic is pre-defined (e.g., χ^2^-distributed). While computing the correlation, they test the value against zero using the pre-defined distribution. The surrogate approach provides an empirically derived null distribution, whereas the approach of Gottman and Ringland [41] would require the selection of a pre-defined distribution that could be used to determine significance of computed correlations. Another approach is to increase the cut-off for the comparison of genuine and pseudosynchrony [42]. This may be realized by introducing a cut-off value for meaningful correlations or *R*^*2*^ values.

### Research questions

In the literature, most of the TSAM are applied with various parameter settings (e.g., transformation, smoothing, method, bandwidth, control for spurious correlations), which are largely based on the researchers’ preferences or theoretical considerations. Apart from Altmann [10] and Paxton and Dale [26], validation studies are lacking evidence. Especially the validation of TSAM against human-rated synchrony would be an important contribution to the field. In the present study, we examine the quality of movement synchrony identification of different TSAM configurations depending on the parameters described, while using human-rated synchronization intervals and simulated time series build out of the human-rated intervals with varying complexity as reference. Aim of this paper is to exemplify a validation of TSAM regarding their capability to correctly identify synchronization intervals given an external criterion (in our case: simulated time series and human ratings of movement synchrony).

## Methods

### Step 1: Selection of video material and generation of time series

To create a dataset of video sequences with and without movement synchrony under naturalistic conditions, 40 sequences showing different amounts of movement synchrony (e.g., synchronous head nodding or body movements which provoke the impression to be interrelated) where selected by the first author. Sequences were extracted from video recordings of the 3^rd^ psychotherapy session of social anxiety disorder patients treated with cognitive-behavioral therapy or psychodynamic therapy. The video recordings originated from the SOPHO-NET treatment study, which was conducted between 2007 and 2009 (for more details see [43, 44]). [for more details, see 43, 44]. Only videos with an optimal camera position and acceptable recording conditions were included. The sequences had a mean duration of 116.69 (*SD* = 11.35) seconds.

All 40 sequences were rated by three independent female raters with a master’s degree in psychology, age range: 26 to 28 years. The raters were familiar with the concept of movement synchrony and were instructed about the general working procedure of the algorithms we use to measure movement synchrony. Before rating, all raters had to rate two example sequences correctly to ensure that they understood the instruction. On a dichotomous scale, the raters were asked to rate whether the sequence showed patient and therapist movements, which were related to each other in any possible way (synchrony: yes) or not (synchrony: no). Rater had no restrictions of the time lag; therefore, they could rate perfectly synchronous *or* time-lagged movements as synchrony. In addition, raters were allowed to watch the sequence as often as they wanted to. Of all video sequences, 18 were rated consistently as sync-sequences and 11 as no sync-sequences. If a sequence was rated as containing movement synchrony, the interval in which this took place was additionally identified (= movement synchronization interval: MSI). Ten movement synchrony sequences and ten sequences showing no movement synchrony, which were rated equally by all raters (sync or no sync), were included for subsequent analysis.

The videos of selected sequences (*N* = 10 with synchrony + 10 without synchrony) were converted into a consistent video format (size of 640x480, frame rate of 25 fps, bit rate of 2000 Kb/s using Any Video Converter 3.0 [45]. Sequences had a mean duration of 118 (*SD* = 12.94) seconds. Patient and therapist movements displayed in the videos were assessed using MEA [46] MATLAB scripts are publicly available at: https://github.com/10101-00001/MEA. The ROI covered the upper body from the chair’s seat upwards. Next, the starting and end points (in frames) of the specified MSI were identified using the plot of the time series. The starting point was defined as the first frame that showed a motion energy value greater than zero proximal to the human-rated starting point. The end point was set proximal to the human-rated end point, where the motion energy time series were equal to zero again. MSI had a mean duration of 145 frames (5.8 seconds), a standard deviation of 57.93 frames (2.37 seconds), a minimum duration of 26 frames (1.04 seconds) and a maximum duration of 282 frames (11.28 seconds). The individuals shown in Fig 1 in this manuscript have given written informed consent (as outlined in PLOS consent form) to publish their case details.

### Step 2: Generation of synchrony conditions

**Fig 4:**
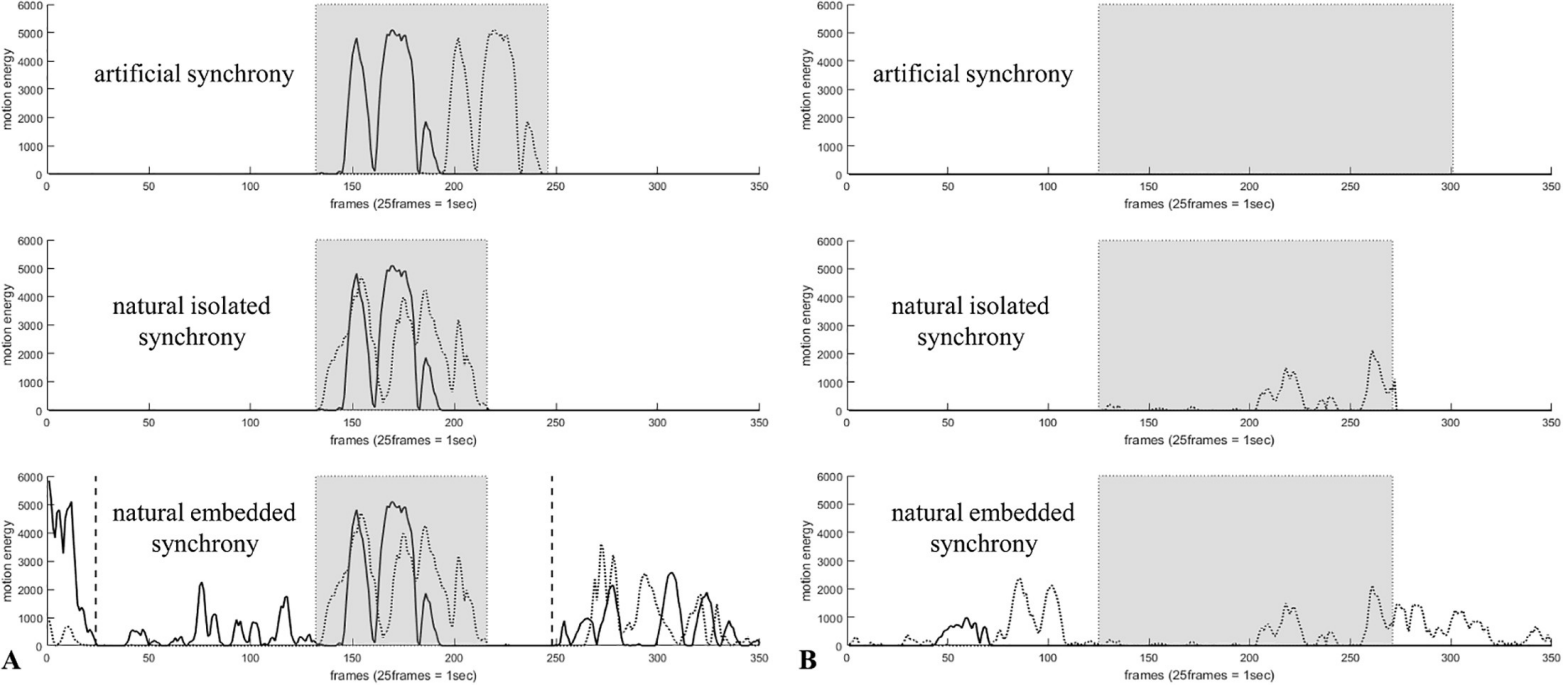
Examples of the three different conditions with an MSI (A) and a no MSI (B) using two time series (patient and therapist movements). Artificial synchrony (upper plot), naturally isolated synchrony (middle), naturally embedded synchrony (lower plot). Gray boxes indicate human rated MSI or no MSI; dashed vertical lines indicate the area investigated by the algorithms.

We used three different experimental conditions since parameter settings should be tested with respect to varying complexity of the stimulus material: 1) natural synchrony rated by humans embedded in a normal interaction, 2) natural synchrony rated by humans isolated from normal interaction, 3) artificial synchrony. In the first condition, patient and therapist METS resulting from MEA were used (see Fig 4, lower figure, gray box indicates MSI within the sequence). Due to the fact that the natural time series were very complex and included other movements apart from the MSI (or no MSI), we modified the time series to obtain a naturally isolated condition: All values of both time series (patient and therapist time series) were set to zero excluding the motion energy values of the MSI (or no MSI) identified by the human raters. Thus, this condition incorporates only the movements of both persons within the specified MSI (or no MSI). Thirdly, we generated an artificial condition: Given the time series of A and B from the naturally isolated condition, we replaced B’s time series with a time-lagged time series of A (time lag of 50 frames). Therefore, we obtained an MSI showing perfect time-lagged movement synchrony. Therefore, in the artificial condition the *true* synchronization interval is known due to the creation of a time series pair. In contrast, in the isolated and natural condition the reference synchronization interval is rated by human raters and can be biased. With respect to the no MSI group, we did not duplicate the patient time series but included a time series of zeros as the therapist time series. If the patient did not move, this therefore resulted in two time series with no movement. The different time series are displayed in Fig 4.

As a result, we obtained 10 naturally embedded synchrony time series pairs, 10 naturally isolated synchrony time series pairs and 10 artificial synchrony time series pairs for MSI and no MSI, respectively (total *n* = 3 (naturally embedded vs. naturally isolated vs. artificial) * 2 (sync vs. no sync) * 10 (video sequences) = 60). Note that the artificial condition can be viewed as simulated time series as they can be easily replicated. We used parts of the real-interaction time series to build those time series, but this was only a matter of choosing those pulses that are realistic for human interactions, particularly realistic for the therapeutic context.

### Step 3: Applying 600 configurations of TSAM algorithms

Next, multiple TSAM with different parameter settings were applied to METS of video sequences. In total, 600 different configurations were investigated by combining method, (WCLC_F_, WCLR), type of transformation (raw data, size standardization, Box-Cox transformation with λ = 0, Anscombe transformation), smoothing (no smoothing, slight smoothing with smoothing splines (λ = .900), high smoothing with smoothing splines (λ = .005)), bandwidth (75 frames (= 3 seconds), 125 frames (= 5 seconds), 175 frames (= 7 seconds), 250 frames (= 10 seconds) and 750 frames (= 30 seconds)) and *R*^2^ cut-off to filter spurious correlations (*R*^2^ > 0.0, *R*^2^ > 0.1, *R*^2^ > 0.2, *R*^2^ > 0.25, and *R*^2^ > 0.3). In the following sections, we omit the ‘F’ of WCLC_F_ for readability. Note that Box-Cox transformation with λ = 0 equals to a logarithmic transformation; thus we increased each motion energy value with 1 to avoid log(0) before applying this transformation.

Assessing the motion energy of two individuals with MATLAB [46] has the advantage that the size of the regions of interest (ROIs) can be saved. This allows the standardization of the METS by the size of the ROI. First, we determined the ratio of the larger ROI to the smaller ROI in the video sequence (range of ROI sizes ratios within a video was 1.03 to 1.99, *M* = 1.37, *SD* = 0.36). Next, we multiplied the ratio with each element of the METS of the individual with the smaller ROI size. The size standardization was used as one possible transformation aside from Box-Cox and Anscombe transformations.

With regard to the automated identification of MSI, we used WCLC and WCLR as implemented by Altmann [10], Altmann [11] and set the maximum time lag to 75 frames (= 3 seconds). Before applying both procedures but after transformation and smoothing, we added some noise (*M* = 0, *SD* = .1) to the time series to make a calculation of a correlation and regression possible. To reduce computation time, values were computed every two frames. Applying one configuration to the time series resulted in an *m* x *n* matrix (*m* = duration of the video sequence in frames/2, *n* = number of time lags/2) of *R*^2^ values. To determine intervals showing the highest *R*^2^ values, peak-picking algorithm of Altmann [10], Altmann [11] was used (MATLAB script publicly available: https://github.com/10101-00001/sync_ident). The output of the algorithm is a list containing every MSI within the video sequence with its time lag, starting, end, and average *R*^2^ value. To account for spurious correlations, we included an *R*^2^ cut-off (*R*^2^ > 0.0, *R*^2^ > 0.1, *R*^2^ > 0.2, *R*^2^ > 0.25 and *R*^2^ > 0.3) to filter MSI of the output list below the cut-off. The data of this study is publicly available at GitHub: https://github.com/DesireeSchoenherr/data_validation_study.

### Step 4: Statistical analysis: Investigation of the best configuration

To determine which configuration identifies synchrony best, we were interested in high power of identification (i.e., valid identification of MSI) and low type I error (i.e., low percentage of over-identified MSI in sequences where no movement synchrony was present). Therefore, a configuration was rated as good if it showed high power in the presence of synchronization intervals and low type I error in their absence. In addition, a configuration was only rated as good if the criteria hold for *each* video sequence.

To compare the ratings with each other, human ratings of the video sequence/true synchronization intervals of the artificial time series were transposed to a time series with zeros and ones, indicating whether movement synchrony was present (= 1) or not (= 0). This time series functioned as a reference time series. In addition, a binary time series of the algorithm was built by setting the time series to one in an automatically identified MSI and to zero outside an MSI. As a measure of concordance of simulated/human and computer-rated synchronization intervals, we computed Cohen’s kappa using both binary time series. A widely established scale for the interpretation of kappa suggests the following cut-offs: values ≤ 0 poor agreement, .00 –.20 slight agreement, .21 –.40 fair agreement, .41 –.60 moderate agreement, .61 –.80 substantial agreement and .81–1.00 almost perfect agreement [47]. With regard to the no MSI group, the proportion of over-identified frames (abbreviated as: pr_out) was calculated that equals the number of identified frames relative to the number of frames in a sequence.

The parameter configuration with the highest identification rate (IR) of synchrony was identified using a sequential procedure: First, the time series in the artificial condition were investigated to ensure that the configurations were able to detect artificial synchrony and no synchrony (high power, low type I error). Configurations with a kappa higher than .60 for *each* of the 10 synchronization intervals (high power) or a maximum value of 5% over-identified frames with respect to *each* of the 10 no synchronization intervals (low type I error) were classified as configurations with a good identification rate (IR). Configurations that yielded a minimum kappa between .41 and .60 for synchronization intervals or 5% to 10% over-identified frames for no synchronization intervals were evaluated as configurations with an acceptable IR. Configurations that did not fulfill either of these criteria were rated as configurations with a poor IR (sync: kappa < .40, no sync: more than 10% pr_out). Based on these classifications, the three IR levels were used to produce cross tables and conduct Fisher’s exact test to check for significant overall influences of the manipulated parameters (e.g., bandwidth, smoothing amount). To examine which specific parameter configuration is best, we additionally conducted ordinal logistic regressions using the function polr in R [48]. Thereby, we used the IR as criterion and the parameters (levels were dummy coded) as predictors. Coefficients of the ordinal logistic regression which are higher than zero indicate that the parameter has a positive influence on the IR (higher kappa, lower pr_out); coefficients lower than zero indicate that the parameter has a negative influence on the IR (lower kappa, higher pr_out). A significant predictor (e.g., method WCLC vs. WCLR) indicates that the IR is affected by the parameter.

Second, we investigated the data with respect to the naturally isolated condition, using *only the configurations that showed a good IR in the artificial condition*. Again, we determined the configurations with a good, moderate and bad IR. In addition, we conducted an ordinal logistic regression with respect to the naturally isolated condition, where only configurations with a good IR in the artificial condition were used.

Third, we examined the data with respect to the naturally embedded condition. Therefore, we used the *configurations with a good or acceptable IR for all video sequences in the naturally isolated condition*.

Lastly, we investigated whether the video sequence had an influence on the validity of identification using two Kruskal-Wallis-tests with the average kappa (kappa_mean_) or the average percentage of over-identified frames (pr_out_mean_) as the dependent variable and the video sequence as independent variables. As our independent variables are not normally distributed (*p* < .001), we used Kruskal-Wallis-test. Note, that a sequence ID only characterizes the stimulus. Therefore, the Kruskal-Wallis-test will show us if there is a dependency between the sequence ID (categorical) and the dependent variables (kappa_mean_, pr_out_mean_, both continuous). However, since the independent variable is categorical, the type of dependency cannot be characterized further.

In additional analyses, we also investigate the general influence of the parameters on the average of kappa and pr_out. Details and results are reported in the Supporting information (S2 Appendix).

## Results

### Artificial condition

Firstly, we considered the artificial condition and counted the number of configurations with a poor, acceptable and good IR depending on their parameter setting (e.g., smoothing, bandwidth, etc.). The corresponding cross table is reported in Table 2.

**Table 2:** Cross table of the variables method, bandwidth, transformation, smoothing, and *R*^*2*^ cut-off value (artificial condition) and identification rate (IR) in synchronization intervals.

		Poor IR		Acceptable IR		Good IR		
		κ < .4	pr_out > 10%	.4 < κ < .6	10% > pr_out > 5%	κ > .6	pr_out < 5%	total
Smoothing	Raw data	42 (21.0%)	49 (24.5%)	110 (55.0%)	15 (7.5%)	48 (24.0%)	135 (68.0%)	200 (33.3%)
	Slight smoothing	41 (20.5%)	47 (23.5%)	131 (65.5%)	17 (8.5%)	28 (14.0%)	136 (68.0%)	200 (33.3%)
	High smoothing	28 (14.0%)	47 (23.5%)	172 (86.0%)	8 (4.0%)	0 (0.0%)	145 (72.5%)	200 (33.3%)
Transformation	Raw data	28 (18.7%)	37 (24.7%)	98 (65.3%)	5 (3.3%)	24 (16.0%)	108 (72.0%)	150 (25.0%)
	Size standard.	26 (17.3%)	35 (23.3%)	108 (72.0%)	7 (4.7%)	16 (10.7%)	108 (72.0%)	150(25.0%)
	Log-trans	26 (17.3%)	36 (24.0%)	104 (69.4%)	13 (8.7%)	20 (13.3%)	101 (67.3%)	150 (25.0%)
	Anscombe	31 (20.6%)	35 (23.3%)	103 (68.7%)	15 (10.0%)	16 (10.7%)	100 (66.7%)	150 (25.0%)
Method	WCLC	66 (22.0%)	132 (44.0%)	202 (67.3%)	0 (0.0%)	32 (10.7%)	168 (56.0%)	300 (50.0%)
	WCLR	45 (15.0%)	11 (3.7%)	211 (70.3%)	40 (13.3%)	44 (14.7%)	249 (83.0%)	300 (50.0%)
Bandwidth	75	0 (0.0%)	48 (40.0%)	100 (83.3%)	8 (6.7%)	20 (16.7%)	64 (53.3%)	120 (20.0%)
	125	0 (0.0%)	48 (40.0%)	100 (83.3%)	8 (6.7%)	20 (16.7%)	64 (53.3%)	120 (20.0%)
	175	1 (0.8%)	12 (10.0%)	83 (69.2%)	11 (9.2%)	36 (30.0%)	97 (80.8%)	120 (20.0%)
	250	5 (4.2%)	17 (14.2%)	115 (95.8%)	7 (5.8%)	0 (0.0%)	96 (80.0%)	120 (20.0%)
	750	105 (87.5%)	18 (15.0%)	15 (12.5%)	6 (5.0%)	0 (0.0%)	96 (80.0%)	120 (20.0%)
*R*^2^ cut-off	> 0.00	27 (22.5%)	71 (59.2%)	91 (75.8%)	28 (23.3%)	2 (2.7%)	21 (17.5%)	120 (20.0%)
	> 0.10	21 (17.5%)	24 (20.0%)	88 (73.3%)	4 (3.3%)	11 (9.2%)	92 (76.7%)	120 (20.0%)
	> 0.20	21 (17.5%)	24 (20.0%)	88 (73.3%)	4 (3.3%)	11 (9.2%)	92 (76.7%)	120 (20.0%)
	> 0.25	21 (17.5%)	0 (0.0%)	58 (48.3%)	0 (0.0%)	41 (34.2%)	120 (100.0%)	120 (20.0%)
	> 0.30	21 (17.5%)	24 (20.0%)	88 (73.3%)	4 (3.3%)	11 (9.2%)	92 (76.7%)	120 (20.0%)

Note. WCLC = windowed cross-lagged correlation, WCLR = windowed cross-lagged regression, bandwidth refers to the frame number. We used a frequency of 25 frames per second for our analysis, size standard. = size standardization, IR = identification rate, cell row proportions in brackets, κ = Cohen’s kappa, pr_out = percentage of over-identified frames.

To investigate the influence of different parameters on the IR, we conducted Fisher’s exact tests because for some cells the assumptions of a *χ*^2^-test were not fulfilled (not more than 20% of the expected counts less than 5, provided that none are less than 1). We found that the choice of method, bandwidth, smoothing amount and *R*^2^ cut-off influenced the IR in the MSI group (smoothing: *p* < .001, Cramer’s V = 0.23; method: *p* = .049, Cramer’s V = 0.10; bandwidth: *p* < .001, Cramer’s V = 0.66; *R*^*2*^ cut-off: *p* < .001, Cramer’s V = 0.24). The highest influence was found for bandwidth, the lowest for method. The frequencies in Table 2 (kappa) suggest that bandwidth should be rather low (175 and/or smaller), a low amount of smoothing should be applied and the *R*^2^ cut-off should be set to 0.25. No significant effects were found with respect to transformation (*p* = .77).

Referring to the artificial no MSI condition (pr_out), we found significant effects for method, bandwidth, and *R*^2^ cut-off (method: *p* < .001, Cramer’s V = 0.37; bandwidth: *p* < .001, Cramer’s V = 0.50; *R*^*2*^ cut-off: *p* < .001, Cramer’s V = 0.48). The largest influence was found for bandwidth, the lowest for the amount of smoothing. The frequencies in Table 2 (pr_out) suggest that bandwidth should be rather high, the WCLR should be applied and the *R*^2^ cut-off should be set to 0.25. No significant effects were found with respect to transformation and smoothing (transformation: *p* = .28; smoothing: *p* = .41).

To identify the best parameter also scientifically, we conducted ordinal logistic regressions. This offers the possibility to also control for other covariates. Results of the ordinal logistic regression based on kappa (log-likelihood = -196.44, *df* = 16) and pr_out (log-likelihood = -214.33, *df* = 16) are displayed in Table 3.

**Table 3:** Results of the ordinal logistic regression with IR (kappaor pr_out) as criterion and parameters as predictors (artificial condition).

	*b* (kappa)	*b* (pr_out)
Smoothing	reference group: smoothing = raw data	
Slight	-0.84*	0.04
High	-1.72*	0.43
Transformation	reference group: transformation = raw data	
Size-standard.	-0.39	0.04
Log-trans	-0.13	-0.36
Anscombe	-0.75	-0.38
Method	reference group: method = WCLC	
WCLR	1.11*	4.14*
Bandwidth	reference group: bandwidth = 75	
125	0.00	0.00
175	0.97*	3.65*
250	-4.00*	3.39*
750	-10.61*	3.34*
*R*^*2*^ cut-off	reference group: *R*^*2*^ cut-off = 0.25	
0.0	-3.80*	-34.54*
0.1	-1.85*	-28.53*
0.2	-1.85*	-28.53*
0.3	-1.85*	-28.53*

Note. WCLC = windowed cross-lagged correlation, WCLR = windowed cross-lagged regression, bandwidth refers to the frame number. We used a frequency of 25 frames per second for our analysis, size standard. = size standardization, IR = identification rate,

* indicates significant associations (*p* < .05), kappa = Cohen’s kappa, pr_out = percentage of over-identified frames.

The coefficients for kappa indicate that transformation do not have an impact on the IR. No smoothing is best in comparison to slight and high amount of smoothing. The WCLR is superior in comparison to the WCLC (reference group). Additionally, a bandwidth of 175 and an *R*^*2*^ cut-off of 0.25 (reference group) seem to be optimal for synchrony detection in MSI within the artificial condition.

The results regarding pr_out show that smoothing and transformation do not have an impact on the IR of over-identified frames. High significant coefficients indicate a positive influence on the IR (lower false positives, lower pr_out). Parameters that were best suited for the identification were the WCLR, a higher bandwidth and an *R*^*2*^ cut-off of 0.25.

Within the artificial condition, 76 configurations fulfilled both criteria for a good IR (kappa > .6 and pr_out < 5%) and therefore showed high power and low type I error. Using these configurations, the average and standard deviation of the rater concordance (minimum kappa, maximum kappa, mean kappa) between the artificially generated movement phenomenon (true MSI) and algorithm ratings of the 10 video sequences containing MSIs were: *M*_kappa_min_ = .64, *SD*_kappa_min_ = .05; *M*_kappa_max_ = .90, *SD*_kappa_max_ = .05, *M*_kappa_mean_ = .77, *SD*_kappa_mean_ = .05. The average and standard deviation of over-identified frames in the no MSI group were: *M*_pr_out_min_ = 0.06%, *SD*_pr_out_min_ = 0.17%, *M*_pr_out_max_ = 1.01%, *SD*_pr_out_max_ = 1.72%, *M*_pr_out_mean_ = 0.32%, *SD*_pr_out_mean_ = 0.70%.

### Naturally isolated and embedded condition

Second, we examined the data in the naturally isolated condition. We found only two configurations with identical identification of MSI and no MSI, which fulfilled our criteria of an acceptable IR. These configurations were *WCLC*, *log-transformed*, *no smoothing*, *R*^*2*^ *cut-off = 0*.*25 with a bandwidth of 75 or 125 frames* (= 3 or 5 seconds). However, we also conducted an ordinal logistic regression to identify the best parameters scientifically. Note that we only used the configurations that were rated as good in the artificial condition; thus some parameter levels are missing. Results are displayed in Table 4 (kappa: log-likelihood = 12.56, *df* = 13; pr_out: log-likelihood = -5.57, *df* = 13). As reference values, we used the best configuration that was identified (WCLC, log-transformed, no smoothing, *R*^*2*^ cut-off = 0.25, bandwidth = 75 frames).

**Table 4:** Results of the ordinal logistic regression with IR (kappa) as criterion and parameters as predictors (naturally isolated condition).

	*b* (kappa)	*b* (pr_out)
Smoothing	reference group: smoothing = raw data	
Slight	-.95	0.00
Transformation	reference group: transformation = log-transformed	
Raw data	-28.70*	0.01
Size-standard.	-63.08*	-6.50
Anscombe	-26.29*	-6.50
Method	reference group: method = WCLC	
WCLR	0.95	9.40
Bandwidth	reference group: bandwidth = 75	
125	0.00	0.01
175	-61.34*	-7.38
*R*^*2*^ cut-off	reference group: *R*^*2*^ cut-off = 0.25	
0.0	-23.57*	-8.97
0.1	-21.06*	-8.06
0.2	-21.06*	-8.06
0.3	-21.06*	-8.06

Note. WCLC = windowed cross-lagged correlation, WCLR = windowed cross-lagged regression, bandwidth refers to the frame number. We used a frequency of 25 frames per second for our analysis, size standard. = size standardization, IR = identification rate,

* indicates significant associations (*p* < .05), kappa = Cohen’s kappa, pr_out = percentage of over-identified frames.

Result of the IR based on kappa showed that the reference configuration is superior to all other parameter settings. However, with respect to the IR based on pr_out there are no differences between different parameter configurations.

In the naturally embedded condition, no configuration was rated as having a good or acceptable IR. In Table 5, means of kappa (MSI) and the average pr_out (no MSI) for the configurations with the best IR of the artificial and naturally isolated conditions as well as the artificial condition only are shown for each of the three time series types (artificial, naturally isolated, naturally embedded). In the naturally embedded time series, the IR is rather low; approximately 50% of frames are over-identified in the no MSI group.

**Table 5:** Cohen’s kappa (MSI) and over-identified frames pr_out (no MSI) of the two best configurations of the artificial and naturally isolated conditions and the best configurations of the artificial condition for all three time series types.

	Sync sequences			No sync sequences		
	*M*_kappa_			*M*_pr_out_		
Configuration	Simu	Nat iso	Nat emb	Simu	Nat iso	Nat emb
Best configurations artificial and naturally isolated synchrony						
WCLC_75_log_nosmooth_0.25	.83	.64	.02	1.20	2.13	57.66
WCLC_125_log_nosmooth_0.25	.83	.64	.02	1.20	2.13	57.66
Best configurations artificial synchrony						
WCLC_75_raw_slight_0.25	.84	.57	-.02	0.50	2.82	44.63
WCLC_75_size_slight_0.25	.84	.57	-.02	0.00	2.82	43.27
WCLC_125_raw_slight_0.25	.84	.57	-.02	0.50	2.82	44.63

Note. *M*_Kappa_ = mean of all stimuli (sequences) with respect to kappa_mean; pr_out = proportion of over-identified frames to all no sync frames; simu = artificial condition/simulated synchrony; nat iso = naturally isolated condition/synchrony; nat emb = naturally embedded condition/synchrony, WCLC = windowed cross-lagged correlation; 75 or 125 indicates bandwidth; raw = raw data; log = logarithmic transformed, size = size-standardized; slight = slight smoothing; 0.25 indicates the *R*^2^ cut-off, WCLC = WCLC_F_.

### Influence of the video sequence

Lastly, we examined the influence of the video sequence on kappa_mean_ and the amount of over-identified frames (pr_out_mean_). We found a significant effect with respect to all three time series types when comparing kappa_mean_ between video sequences (artificial: *χ*^*2*^ = 520.47, *df* = 9, *p* < .001; naturally isolated: *χ*^*2*^ = 552.81, *df* = 9, *p* < .001; naturally embedded: *χ*^*2*^ = 717.54, *df* = 9, *p* < .001). Ranks of the sequences are displayed in S1 Table. For example, video sequence number 7 was the sequence with a very low kappa with respect to the naturally isolated condition. However, it was the best sequences referring to the artificial and naturally embedded condition. We also found a significant effect with respect to two time series types when comparing pr_out_mean_ between video sequences (naturally isolated: *χ*^*2*^ = 1631.10, *df* = 9, *p* < .001; naturally embedded: *χ*^*2*^ = 817.70, *df* = 9, *p* < .001). There were no differences for the artificial condition (*χ*^*2*^ = 6.13, *df* = 9, *p* = .73). For example, video sequence 1 was best with respect to the naturally isolated condition, whereas for the naturally embedded condition video sequence number 15 was best. Since we do not have any variables describing these sequences further, it remains an open question which characteristics of a video sequence are important for being a good sequence.

## Discussion

The aim of this study was the validation of TSAM regarding their capability to correctly identify synchronization intervals given an external criterion. The peak-picking algorithm in combination with the WCLC or WCLR of Altmann [10], Altmann [11] allows the identification of MSI so that predefined synchronization intervals (e.g., rated by a human rater or artificially generated by a time series simulation) can be compared with MSI identified by TSAM. We tested the validity of varying configurations with MSI and no MSI to find a configuration with high power (correct positives) and low type I error (false positives) with respect to the identification of MSI. Former studies having tested TSAM or varied parameter settings are rare. Ramseyer and Tschacher [49] showed that synchrony is dependent on the time lag and segment size by using different parameter settings in a case study. Altmann [10], Altmann [11] tested the WCLC and WCLR by using simulated time series in a case study. Therefore, the current study contributes to the examination of the impact of different parameter settings on the identification of movement synchrony by using n = 60 time series pairs.

### Artificial condition

Our results show a clear influence of method, bandwidth, smoothing amount and *R*^2^ cut-off on the identification rate (IR) in MSI. These parameters, except for the smoothing amount, also affect the frequency of over-identified frames in the no MSI condition. The WCLR is significantly superior to the WCLC. A bandwidth of 175 frames (= 7 seconds) leads to the best results, followed by smaller bandwidths such as 75 or 125 frames (= 3 and 5 seconds) regarding to the MSIs. With regard to smoothing, it is advisable to use raw data or to smooth the data slightly at most. An *R*^2^ cut-off of 0.25 provides the best IR for MSI and no MSI.

For the subsequent analyses, we excluded configurations showing an acceptable or poor IR in the artificial condition. Of all *N* = 600 configurations within the artificial condition, there were 76 that displayed a good IR (κ > .6, pr_out < .05) for each of the video sequences. On average, these configurations had a very high inter-rater level of agreement between artificially generated MSI and computer-based ratings and a small proportion of over-identified frames (*M*_kappa_mean_ = .77, *SD*_kappa_mean_ = .05, *M*_pr_out_mean_ = 0.32%, *SD*_pr_out_mean_ = 0.70%). This indicates that the presented methods are able to detect perfect synchrony with a constant time lag (also known as echoing). The influences of specific parameters are discussed in detail in the following.

A parameter that affected the IR of synchrony was the amount of smoothing. So far, different smoothing amount have not yet been compared. The graphics in the introduction illustrate that if the time series does not contain any errors due to video defects, it is quite plausible to not smooth the data too much to avoid shifting or erasing peaks. Accordingly, our results suggest that no smoothing or slight smoothing is advisable. If the time series contains such errors, filtering is important to avoid biases in MSI identification.

Transformation appears to have no significant influence on the identification of MSI. Transformations are performed to achieve stationarity and to transform the distribution into an approximately normal distribution [10]. The use of windows in the calculation of the MSI was also implemented to ensure local stationarity. It appears that the use of the windows is sufficient to deal with distribution-related challenges and there is no need for a stabilizing transformation.

Regarding the method used (WCLC vs. WCLR) we found a significant effect in the artificial condition. The WCLR showed higher kappas and lower false positives than the WCLC. The effect size was small to medium (Cramer’s V = 0.10–0.37) in favor of WCLR. The difference between WCLC and WCLR may be explained by the fact that the WCLR was developed for cyclic or auto-correlated time series.

With regard to bandwidth, we found that rather small bandwidths lead to good results. Additionally, bandwidth seems to have the highest impact on results. Ramseyer and Tschacher [49] reported high synchrony values with bandwidths of 60 to 90 seconds (i.e., 600 to 900 frames). These contradictory results can be explained by the different synchrony measure and research question. While Ramseyer and Tschacher [49] calculated the strength of synchrony of the total interaction [17], our algorithms measured the frequency of synchrony [10, 17]. Moreover, while Ramseyer and Tschacher [49] classified the highest cross-correlation in comparison to surrogate-based pseudosynchrony, they did not compare the localization of a synchronization interval by a human rater/simulated MSIs to the identification by TSAM. Therefore, the criterion against which synchrony is compared differs: for Ramseyer and Tschacher [5] it is synchrony measured in surrogate datasets, in the current study it is either simulated perfect/imperfect synchrony, or synchrony rated by human raters. In addition, the different stimulus material has an influence on the results, as shown in our study (Ramseyer and Tschacher [49]: split-screen, two cameras; our scenario: naturalistic therapy sessions, one camera).

We introduced an *R*^2^ cut-off to further control for spurious correlations. Our results suggest that an *R*^2^ cut-off improves the IR. The multiple executions of the correlations or regressions to form the *m* × *n R*^2^ matrix (*m* denotes the number of time points in frames, *n* indicates the number of time lags in frames) seem to have the effect that random intervals are identified as synchronous intervals. An increase of the *R*^2^ cut-off can be understood as controlling for such spurious correlations. Note that this procedure is only valid with high true correlations and may be problematic if synchrony is weak.

### Best configuration in the naturally isolated and artificial conditions

**Fig 5:**
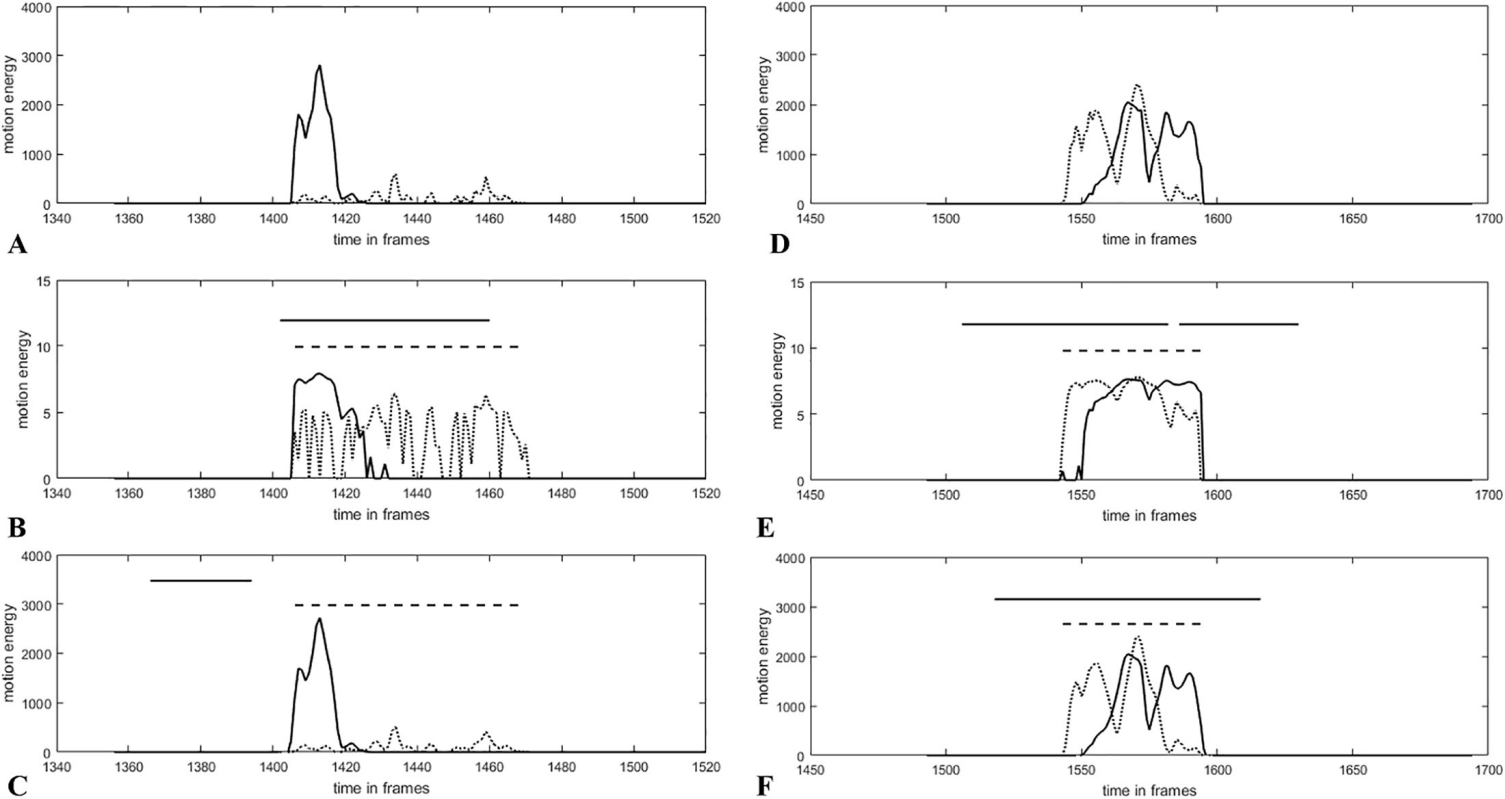
Time series (patient and therapist movements) of the naturally isolated condition. Solid horizontal lines indicate the computer-based synchrony interval (WCLC, bandwidth 125, R^2^ > 0.25); dashed horizontal lines indicate the human-rated synchrony interval of the video sequence. (A) Time series of human-rated synchronization interval with different peak heights, (B) logarithmically transformed time series shown in A, (C) slightly smoothed and not transformed time series shown in A, (D) time series of human-rated synchronization intervals with similar peak heights, (E) logarithmically transformed time series shown in D, (F) slightly smoothed and not transformed time series shown in D.

For further analyses, we only included configurations that had been classified as good in the artificial condition. Investigating the naturally isolated condition, we found only two configurations with an acceptable IR (WCLC, logarithmically transformed, not smoothed, *R*^2^ > 0.25, bandwidth of 75 or 125 frames). It is noticeable that both configurations use logarithmically transformed data, seemingly contradictory to the results in the artificial condition. This inconsistency, however, can be explained as follows: The plots of the time series with identified synchronization intervals (Fig 5) display that the peaks have different shapes in the naturally isolated condition in contrast to the artificial condition. Therefore, a large movement can occur synchronously with a rather weak movement. This difference in the peak heights is matched by the logarithmic transformation and thus allows a better identification of the synchronization intervals compared to a time series that has not been logarithmically transformed (Fig 5, Plot A, B, C). If both time series have approximately the same peak heights, the results differ only slightly (Fig 5, Plot D, E, F). In line with this, the results of the ordinal logistic regression show that the log-transformation is superior to other transformations in MSI detection, provided that the IR is acceptable for all videos. If the general influence of the parameters is investigated (see Supporting information, S2 Appendix), also the Anscombe transformation has a significant influence on the results. Therefore, we recommend using logarithmically transformed time series (or another variance-stabilizing transformation) for the identification of synchrony (based on human-rated synchronization intervals).

For bandwidth, the results point towards the preference of lower bandwidths (75 or 125) as indicated by both best configurations.

Another explanation for the differences between artificial and naturally isolated time series is that the characteristics of the reference material differ substantially. In the artificial condition, a synchronization interval is created as echoing of a given time series part so that the time series of both persons are perfectly equal with a predefined time lag. In contrast, in the naturally embedded configuration the “true” synchronization interval (respectively its beginning and end) is rated by human raters. Our study revealed that a lot of algorithms with very high identification quality in the artificial configuration failed in the naturally embedded configuration. This could mean that the algorithms had another synchrony concept than the human raters in our study. Furthermore, it should be noted that the algorithms and human raters had different levels of information about the nonverbal interpersonal interaction under study. Whereas the algorithms “decided” about the similarity of time series parts of an aggregated movement measure (the motion energy), the decision of a human rater refers to the information in the video including gaze behavior or direction of movement (which were not available for the algorithms). On that reason, future studies should examine simulated stimulus material whereby the time-lag and the shape of the echoed time series are modified. The big advantage of simulated time series is that the synchronization intervals can be defined.

### Naturally embedded condition

**Fig 6:**
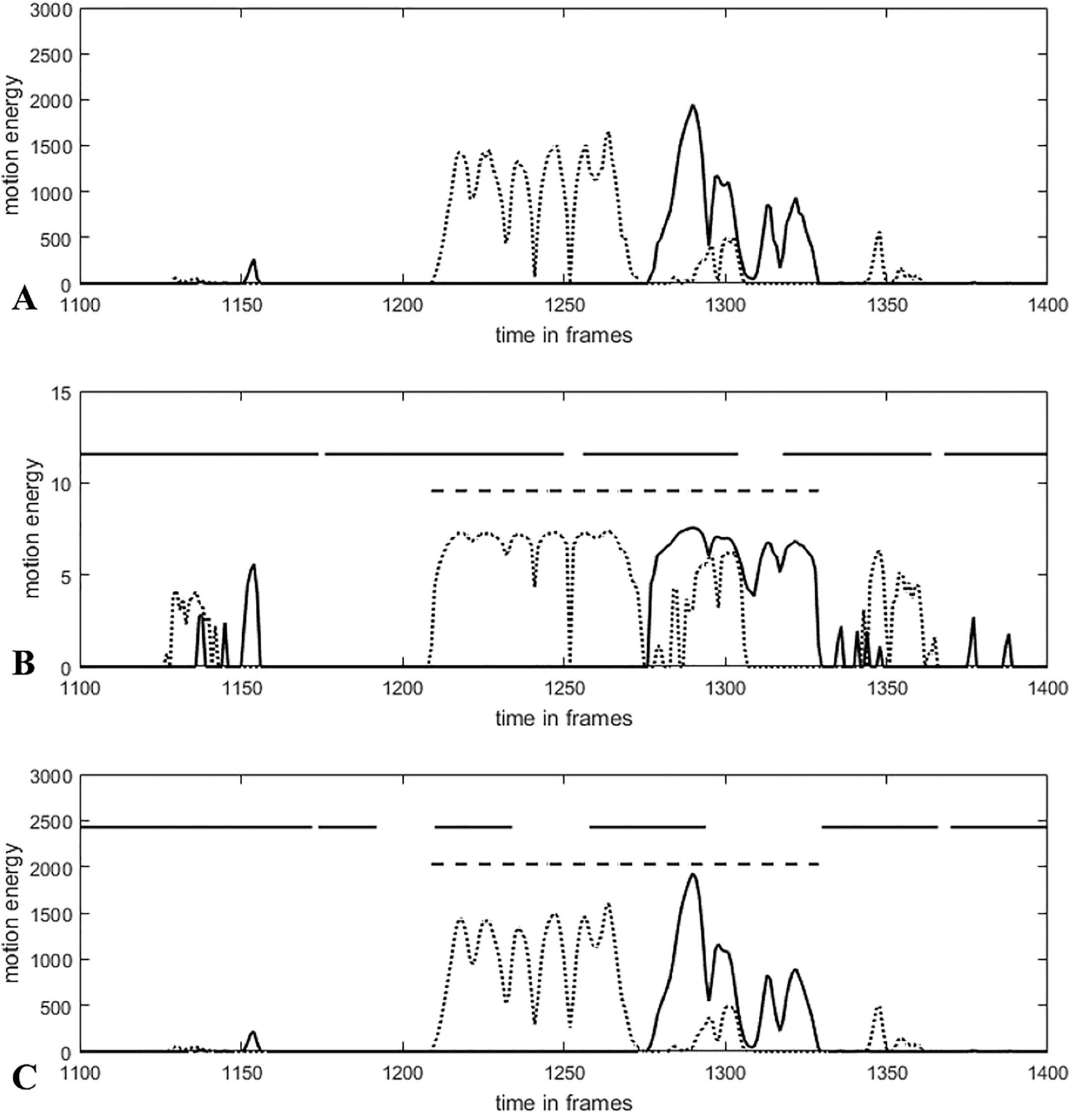
Time series (patient and therapist movements) of the naturally embedded condition. Solid horizontal lines indicate the computer-based synchrony interval (WCLC, bandwidth 125, R^2^ > 0.25), dashed horizontal lines indicate the human-rated synchrony interval of the video sequence. (A) Time series of the human-rated synchrony phenomenon, (B) logarithmically transformed time series shown in A, (C) slightly smoothed and not transformed time series shown in A.

With respect to the naturally embedded time series, we did not find a configuration that reached a kappa of > .4 for all sequences. A reason for this finding may be that the rating of the synchronization intervals was carried out by three human raters who were really similar to each other. Therefore, the construct to which the algorithm-construct of synchrony was compared to was synchrony as rated by three young female psychologists. Movement synchrony was identified when there was an obvious relationship between the movements of both persons interacting. However, the construct measured by the computer-based methods disregards the context of the displayed movements. Only simultaneous or slightly time-delayed movements of the interaction partners are identified as synchrony. That is, an interval in which the therapist is looking for something in a folder and the patient scratches his head would be identified as synchrony by methods, but not by human raters. Fig 6 clearly displays the difference in ratings. The phenomenon between approx. 1125 frames and 1160 frames as well as the phenomenon between 1340 frames and 1360 frames are evaluated as not synchronous by the raters, but are nevertheless identified by the methods, leading to low rater agreement between human rating and computer rating. A possible solution to reduce the over-identification of frames could be to smooth small deflections (Fig 6, *t* = 1375–1400 frames) by applying a moving median (bandwidth of 5 values) [46], or to modify the peak-picking algorithm. Studies using a more representative sample of raters may also be requested. However, Bernieri, Reznick [39] showed that even with a large number of raters (n = 20), ratings are not very reliable. We used similar raters to not additionally include a rating bias based on gender or age because studies show an influence in synchrony (respectively perceiving synchrony) for these variables [50, 51]. Moreover, calculating correlations makes the assumption of a linear relationship between both time series. Allowing for other dependencies may also result in an improved detection of synchrony [52].

Another important aspect to consider is the output of the MEA. MEA aggregates all movements displayed in a video. Therefore, movements of arms, torso and head are aggregated to one movement index. A human rater can differentiate between these different body parts and also considers gaze direction and may therefore understand movement synchrony differently. Moreover, our motion analysis was based on 2-D video recordings and can only partially represent 3-dimensional movements. Another possibility for future research projects is the usage of 3-D cameras such as the Microsoft Kinect.

To summarize, we identified 76 of 600 configurations that allow a good identification of artificially generated MSI and a low false identification rate with respect to artificially generated no MSI. These configurations were found in the artificial condition. In the artificial condition, the time series pair was generated with perfect (time-delayed) synchrony. Therefore, 76 were able to identify perfect echoing with high concordance. These configurations used a low amount of smoothing, the WCLC or WCLR, an *R*^2^ cut-off of 0.25 and rather low bandwidths. Furthermore, we were able to show that computer algorithms identify synchrony in a different way than humans, that is they detect similarity of time series with a good identification rate (artificial condition), but not multimodal synchrony within a context as rated by humans in the present study.

### Limitations & future research

To our knowledge, this study is the first testing parameter settings with respect to time series analysis methods in a validation study using n = 60 time series pairs and 600 different parameter configurations. Therefore, the current study contributes to the investigation of the validity of automated algorithms for the identification of nonverbal synchrony.

One limitation of the present study lies in the fact that we defined the *true* MSI by ratings of similar human raters. It is possible that different instructions and an extended training are required to better capture the construct. Although rater were instructed and familiar with the concept of movement synchrony, human raters have an internal model of synchrony that likely includes keeping eye contact and signals of other modalities that are not assessed by the computer-assisted methods for automated synchrony identification. The resulting difference could be reduced by further instructions or an extended rater training with regard to the concept of movement synchrony. However, it should be kept in mind that all algorithms (parameter configurations) studied in the naturally isolated and naturally embedded condition (in which human ratings were used as reference) were tested and approved by the good IR in the artificial condition. In the artificial condition, the true synchronization intervals were created by programming / simulation and are thus largely unbiased.

Furthermore, the time lag of synchrony was not systematically varied in the stimulus material. However, it is possible that time-lag has an impact on the IR. In a further study, it would also be important to systematically vary the time lag and more finely grade the parameters.

We were able to show that the stimulus material had a significant impact on the IR. This means that generalizability to other study designs is restricted. Further, in future studies, the amount of stimulus material should be increased. Our video sequences had specific characteristics (e. g., therapy videos of patients with social anxiety disorder, one camera setting) and a specific format (size: 640 x 480, etc.). The creation of a publicly accessible video database with annotations of synchronous movements could make a great contribution to the further development of TSAM. With the help of such a database, methods could be validated and the construct of nonverbal synchrony could be defined more explicitly. Such a database would also provide the opportunity to further investigate video characteristics and parameter settings.

### Conclusion

Numerous studies have demonstrated that several problems occur with human ratings (e.g., reduction number of values of the assessed behavior, because of the rater’s capacity, problems defining an equal construct between raters) and that the reliability of human raters is weak [53]. Additionally, human ratings require more time and personal resources and training seems to be important to ensure that all raters assess the same construct. In contrast, automated synchrony identification is economic and reliable. With our study, we demonstrated that the choice of parameter settings is essential for the generation of meaningful results using automated methods for synchrony detection. The tests of the artificial condition suggested that WCLR without smoothing, small bandwidths (75 or 125 frames) and an *R*^*2*^ cut-off of 0.25 is best suited for the identification of perfect echoing. With respect to the naturally isolated condition, we identified WCLC with a bandwidth of 75 or 125 frames (3 or 5 seconds, respectively), no smoothing/slight smoothing, but a logarithmically transformed time series and an additional *R*^2^ cut-off of 0.25 as the best configuration to detect the interpersonal synchrony of body movements (given acyclic time series and MSI showing two different complex peaks). This configuration also showed a good IR regarding high correct positives and low false positives in the artificial condition. In addition, we found low concordance in the naturally embedded condition due to the fact that human raters assessed movement synchrony differently than the applied configurations. Human raters rate synchronous movements by including variables such as eye contact and context variables, resulting in another concept of synchrony. Movement synchrony of our best algorithm goes beyond this definition by assessing all simultaneous or slightly time-delayed highly correlated time series trajectories. Thus, unconscious processes resulting in movement synchrony, which may also have an influence on the therapeutic process, are additionally assessed.

## Supporting information

S1 Appendix Influence of the video sequence. (DOCX)

S2 Appendix General influence of the parameters in all conditions. (DOCX)

S1 Table Ranking of the different video sequences based on Kruskal-Wallis-tests. (DOCX)

S2 Table Coefficients and significance of the ordinal logistic mixed effects regression (criterion IR by kappa, MSI) for all conditions. (DOCX)

S3 Table Coefficients and significance of the ordinal logistic mixed effects regression (criterion IR by pr_out, noMSI) for all conditions. (DOCX)
