# Supplementary material for: Identification of movement synchrony: Validation of windowed cross-lagged correlation and -regression with peak-picking algorithm
Source: PLoS One. 2019 Feb 11;14(2):e0211494. doi: 10.1371/journal.pone.0211494 (PMC6370201; doi:10.1371/journal.pone.0211494)
Supplement: S1 Appendix — (DOCX) [file pone.0211494.s001.docx]

**Influence of the video sequence**

Table in S1 Table displays the ranks of the different video sequences based on Kruskal-Wallis-test. Note that for pr_out in the artificial condition, Kruskal-Wallis-test indicates no significant differences between the video sequences.
