# Supplementary material for: Identification of movement synchrony: Validation of windowed cross-lagged correlation and -regression with peak-picking algorithm
Source: PLoS One. 2019 Feb 11;14(2):e0211494. doi: 10.1371/journal.pone.0211494 (PMC6370201; doi:10.1371/journal.pone.0211494)
Supplement: S2 Appendix — (DOCX) [file pone.0211494.s002.docx]

**General influence of the parameters in all conditions**

To investigate the general influence of the parameters in all conditions by using all videos, we conducted ordinal logistic regressions with a random effect for the video sequence for all three conditions. Results are displayed in S2Table (criterion IR by kappa, MSI) and S3Table (criterion IR by pr_out, noMSI). The difference to the analyses reported in the results section is that we here consider the IR based on the average kappa and pr_out. Therefore, the *average kappa* has to be >.6 for a good identification rate whereas with respect to the analyses in the results section a configuration is rated as having a good IR if each video has a kappa >.6 (*minimum kappa* >.6).

We found that IR was affected by method, degree of smoothing and *R²* cut-off. The WCLR, no smoothing, a variance-stabilizing transformation (Anscombe or log transformation), and *R²* cut-off of 0.25 are superior. Furthermore, a bandwidth of 175 showed a better IR followed by bandwidth of 75 and 125. In each condition, the variance of random intercept was significant. This indicates that the IR depends on the video sequence. Moreover, we observed that the threshold for a moderate IR and good IR increased from artificial to isolated condition as well as from isolated to naturalistic condition. This means that a moderate and good IR is reached less often in the naturalistic condition than for example in the artificial condition. The thresholds of the regression are in line with the values of kappa and pr_out shown in Table 5. The more complex the time series is (artificial < naturally isolated < naturally embedded), the higher are the thresholds. Therefore, fewer configurations reach a high kappa and a low pr_out. In line with this, the amount of pr_out increases while the kappa decreases with increasing complexity of the time series condition.

Our general analyses showed that the configuration that is best is largely dependent on the stimulus material and the experimental condition. Introducing interactional terms may account for the inconsistencies between the different conditions. However, a low amount of smoothing, a variance-stabilizing transformation (e.g., log-transformation or Anscombe-transformation), the WCLR, not too high bandwidths, and an *R²* cut-off of 0.25 seem to influence the identification rate positively. Note that these parameters are best, provided that not all video sequences showed a good IR.
