# Supplementary material for: Identification of movement synchrony: Validation of windowed cross-lagged correlation and -regression with peak-picking algorithm
Source: PLoS One. 2019 Feb 11;14(2):e0211494. doi: 10.1371/journal.pone.0211494 (PMC6370201; doi:10.1371/journal.pone.0211494)
Supplement: S1 Table — (DOCX) [file pone.0211494.s003.docx]

**Table S1. Ranking of the different video sequences based on Kruskal-Wallis-tests.**

|  | Artificial condition rank | Naturally isolated condition rank | Naturally embedded condition rank |
| --- | --- | --- | --- |
| Sequence ID |  |  |  |
|  | Synchrony sequences - kappa | | |
| 3 | 8 | 8 | 2 |
| 4 | 10 | 7 | 5 |
| 7 | 1 | 1 | 9 |
| 8 | 9 | 9 | 7 |
| 10 | 3 | 4 | 10 |
| 13 | 6 | 10 | 6 |
| 14 | 5 | 6 | 1 |
| 17 | 7 | 3 | 8 |
| 18 | 2 | 2 | 4 |
| 20 | 4 | 5 | 3 |
|  | No synchrony sequence - pr_out | | |
| 1 | 1 | 1 | 4 |
| 2 | 7 | 5 | 6 |
| 5 | 8 | 6 | 8 |
| 6 | 2 | 10 | 2 |
| 9 | 6 | 8 | 3 |
| 11 | 10 | 4 | 5 |
| 12 | 3 | 3 | 9 |
| 15 | 5 | 2 | 1 |
| 16 | 4 | 9 | 7 |
| 19 | 9 | 7 | 10 |
