# Supplementary material for: Identification of movement synchrony: Validation of windowed cross-lagged correlation and -regression with peak-picking algorithm
Source: PLoS One. 2019 Feb 11;14(2):e0211494. doi: 10.1371/journal.pone.0211494 (PMC6370201; doi:10.1371/journal.pone.0211494)
Supplement: S2 Table — (DOCX) [file pone.0211494.s004.docx]

**Table S2. Coefficients and significance of the ordinal logistic mixed effects regression (criterion IR by kappa, MSI) for all conditions.**

| Parameter | Artificial condition | Naturally isolated condition | Naturally embedded condition |
| --- | --- | --- | --- |
| Method | reference group: method= WCLC | | |
| WCLR | 2.33^*^ | 1.51^*^ | 0.64^*^ |
| Smoothing | reference group: smoothing = raw data | | |
| Slight | -0.11 | 0.09 | -0.18 |
| High | -1.53 | -0.00 | -0.25^+^ |
| Transformation | reference group: transformation = raw data | | |
| Size-standardization | -0.11 | 0.08 | -0.01 |
| Log-transformation | 0.16^+^ | 0.27^*^ | -0.40^*^ |
| Anscombe-transformation | 0.39^*^ | 0.27^*^ | 0.25^+^ |
| Bandwidth | reference group: bandwidth = 75 | | |
| 125 | -0.00 | -0.00 | -0.00 |
| 175 | 1.92^*^ | -0.05 | 0.15 |
| 250 | 0.18^*^ | -0.64^*^ | 1.17^*^ |
| 750 | -7.89^*^ | -7.82^*^ | -0.27 |
| *R²* cut-off | reference group: *R²* cut-off = 0.0 | | |
| 0.1 | 1.72^*^ | 0.84^*^ | 0.09 |
| 0.2 | 1.72^*^ | 0.84^*^ | 0.09 |
| 0.25 | 3.36^*^ | 1.92^*^ | -0.05 |
| 0.30 | 1.72^*^ | 0.84^*^ | 0.09 |
| Var (u_0_) | 0.81^*^ | 0.48^*^ | 3.38^*^ |
| Thresholds |  |  |  |
| bad \| ok | 0.54 | 1.77^*^ | 4.16^*^ |
| ok \| good | 2.89^*^ | 3.77^*^ | 5.42^*^ |

Note. * indicates significant results on a 5% alpha level, ^+^ indicates significant results on a 10% alpha level, WCLR = Windowed cross-lagged regression.
