# Supplementary material for: Identification of movement synchrony: Validation of windowed cross-lagged correlation and -regression with peak-picking algorithm
Source: PLoS One. 2019 Feb 11;14(2):e0211494. doi: 10.1371/journal.pone.0211494 (PMC6370201; doi:10.1371/journal.pone.0211494)
Supplement: S3 Table — (DOCX) [file pone.0211494.s005.docx]

**Table S3. Coefficients and significance of the ordinal logistic mixed effects regression (criterion IR by pr_out, noMSI) for all conditions.**

| Parameter | Artificial condition | Naturally isolated condition | Naturally embedded condition |
| --- | --- | --- | --- |
| Method | reference group: method= WCLC | | |
| WCLR | 3.29^*^ | 1.95^*^ | 0.57^*^ |
| Smoothing | reference group: smoothing = raw data | | |
| Slight | -0.00 | -0.04 | -1.00^*^ |
| High | 0.36^*^ | -0.70^*^ | -1.93^*^ |
| Transformation | reference group: transformation = raw data | | |
| Size-standardization | 0.02 | -0.10 | -0.05 |
| Log-transformation | -0.08 | 0.37^*^ | 0.26^*^ |
| Anscombe-transformation | -0.06 | 0.37^*^ | 0.16 |
| Bandwidth | reference group: bandwidth = 75 | | |
| 125 | 0.00 | 0.00 | -0.00 |
| 175 | 1.82^*^ | 0.36^*^ | 0.96^*^ |
| 250 | 1.70^*^ | -0.21^*^ | 1.99^*^ |
| 750 | 1.97^*^ | -1.45^*^ | 4.41^*^ |
| *R²* cut-off | reference group: *R²* cut-off = 0.0 | | |
| 0.1 | variable was removed because model did not converge due to empty cells for 0.25 x bad, ok | 1.60^*^ | 1.40^*^ |
| 0.2 |  | 1.60^*^ | 1.40^*^ |
| 0.25 |  | 3.22^*^ | 2.87^*^ |
| 0.30 |  | 1.60^*^ | 1.40^*^ |
| Var (u_0_) | 0.03^*^ | 1.90^*^ | 1.12^*^ |
| Thresholds |  |  |  |
| bad \| ok | -0.10 | 1.18^*^ | 5.09^*^ |
| ok \| good | 0.78^*^ | 3.27^*^ | 5.50^*^ |

Note. * indicates significant results on a 5% alpha level, ^+^ indicates significant results on a 10% alpha level, WCLR = Windowed cross-lagged regression, Var (u_0_) = Variance of the random intercept.
